# Changes in smoking behavior among victims after the great East Japan earthquake and tsunami

**DOI:** 10.1186/s12199-020-00858-5

**Published:** 2020-06-11

**Authors:** Yoneatsu Osaki, Hitoshi Maesato, Ruriko Minobe, Aya Kinjo, Yuki Kuwabara, Aya Imamoto, Yoshinori Myoga, Sachio Matsushita, Susumu Higuchi

**Affiliations:** 1 Division of Environmental and Preventive Medicine, Department of Social Medicine, Faculty of Medicine, Tottori University, 86 Nishi-cho, Yonago, Tottori, 683-8503 Japan; 2 National Hospital Organization Kurihama Medical and Addiction Center, 5-3-1 Nobi, Yokosuka, Kanagawa 239-0841 Japan; 3 Department of Pediatrics, Tottori University Hospital, 36-1 Nishi-cho, Yonago, Tottori, 683-8504 Japan

**Keywords:** Natural disaster, Smoking, Tsunami, Nicotine dependence, Great East Japan earthquake

## Abstract

**Background:**

In areas affected by the tsunami of the great East Japan Earthquake, smoking behavior may have deteriorated due to high stress and drastic changes in living environment. Surveys were conducted to reveal changes in smoking behaviors among victims.

**Methods:**

A population-based random-sample home-visit interview survey of victims in Iwate and Miyagi Prefectures affected by the tsunami disaster was conducted in 2012 (*n* = 1978), while a population-based nationwide survey was conducted in 2013 (*n* = 1082). A panel survey in 2014 was conducted with respondents of the 2012 survey (*n* = 930). Multiple logistic regression analysis was performed to reveal factors related to smoking status after the disaster.

**Results:**

There was high smoking prevalence of both sexes in the tsunami disaster area (current smoking rate in coastal area, 50.0% for male, 21.4% for female; inland area, 34.7% for male, 7.6% for female). Low prevalence of male quitters was observed (quitter rate in coastal area, 20.8% for male, 8.0% for female; inland area, 23.4% for male, 5.5% for female). The prevalence of nicotine-dependent people assessed by FTND (Fagerström Test for Nicotine Dependence) in the coastal area was also higher than in the inland area or other areas of Japan. Smoking behavior among victims worsened after the disaster and did not improve 3 years from the disaster. Post-disaster factors related to smoking were living in coastal area, complete destruction of house, and living in temporary housing.

**Conclusions:**

Smoking prevalence and the level of nicotine dependence of tsunami victims were still high even 3 years after the disaster. It is important to emphasize measures for smoking control in the disaster areas for an extended time period.

## Introduction

The great East Japan Earthquake was a magnitude 9.0–9.1 (Mw) undersea megathrust earthquake off the coast of Japan that occurred on March 11, 2011. It was the most powerful earthquake ever recorded in Japan. The earthquake triggered powerful tsunami waves that may have reached heights of up to 40.5 m (133 ft), and which, in the Sendai area, traveled up to 10 km inland. The tsunami swept the Japanese mainland and killed many people, mainly through drowning, though blunt trauma also caused many deaths. The latest report from the Japanese National Police Agency confirmed 15,897 deaths, 6157 injured cases, and 2533 missing cases across twenty prefectures (as of March 8, 2019) [1], and the number of refugees was approximately 347 thousand at its peak in 2012. A 2019 report indicated that approximately 52,000 people were still living away from their homes in temporary housing [2]. The National Police Agency report listed 121,990 buildings as “totally collapsed,” with a further 282,900 buildings “half collapsed” and another 730,044 buildings “partially damaged” [1]. The earthquake and tsunami also caused extensive, severe infrastructural damage in north-eastern Japan. In the 65 years since the end of World War II, this has been the toughest crisis faced by Japan.

In the disaster area, many people were forced to live long term as evacuees in environments different from those of conventional life, such as temporary housing or rental houses. How health-related lifestyle changed after the disaster is important to understand in order to protect the health of the victims. Some reports have indicated that the smoking behavior of victims has changed after natural and human-made disasters: the September 11, 2001, attacks [3–5]; Hurricane Katrina, in 2005 [6, 7]; bushfires around Canberra, in 2003 [8]; and the Enschede fireworks disaster in the Netherlands, in 2000 [9]. However, there are few reports about smoking behavior after an earthquake or tsunami. Some articles on smoking behavior after a New Zealand earthquake have been published [10], and one article reported decreased smoking prevalence among victims in Fukushima Prefecture after the great East Japan Earthquake [11]. However, no article describing smoking behavior among victims in tsunami-damaged parts of Miyagi and Iwate Prefectures has been found. We conducted a survey to identify changes in smoking behavior of victims after the disaster in Miyagi and Iwate Prefectures. The current study hypothesis was that the smoking status of people in the tsunami-damaged area had worsened after the disaster and that had improved subsequently. The study provides findings that stress the importance of improvising measures for smoking control in disaster areas in the long term to reduce future health hazard.

## Materials and methods

**Fig. 1:**
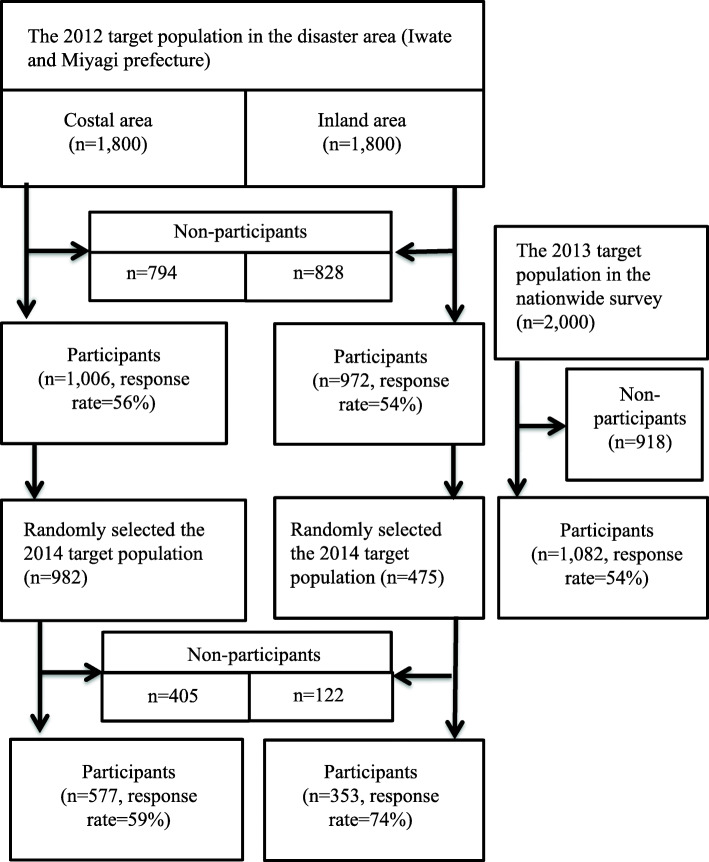
Flow chart for this survey

A population-based random-sample home-visit interview survey of victims in Iwate and Miyagi Prefectures affected by the tsunami disaster was conducted in 2012 (*n* = 1978). In order to compare with the results of the 2012 survey, we conducted a nationwide survey in 2013 except for the three affected prefectures using the same questionnaire (*n* = 1082). A panel survey in 2014 was conducted with respondents of the 2012 survey (*n* = 930). The outline of this study was shown in Fig. 1.

### Participants

Participants of the present survey were survivors living in the disaster area. This study includes an interview survey conducted in the disaster area in 2012, a panel survey in the disaster area 2 years later and a nationwide survey excluding the disaster area in 2013. The survey in the tsunami disaster area was conducted in the Iwate and Miyagi Prefectures. Fukushima Prefecture was excluded from the survey area, because conducting home visits to administer the interview survey was still difficult at that time, and many people were still living as refugees, distant from their registered addresses. A municipality that had a coastline at the boundary was defined as a coastal area. A municipality that did not have a coastline at the boundary was defined as an inland area. Sendai City of Miyagi Prefecture, which has a large population, defined a ward with coastline at the boundary as a coastal area and a ward without the coastline as an inland area.

### Procedures

We randomly selected 1800 people from the coastal area and 1800 people from the inland area in the Iwate and Miyagi Prefectures using the resident resisters of local municipalities. Trained investigators requested them for their cooperation and visited participants who had consented to an interview. We entrusted a survey company called the Shin Joho Center to carry out the sample selections and home visit interview surveys. The survey company requested the sampling of residents to the municipalities, and the investigators visited municipal offices and randomly selected residents from the Basic Resident Register. Investigators were 49 well-trained employees of the survey company living in Miyagi and Iwate Prefectures.

The number of respondents was 1006 for the coastal area (56% of response rate and 58% of actual response rate excluded by moving, address unknown, and long-time absent) and 972 for the inland area (54% of response rate, 59% of actual response rate). The survey period was November to December 2012.

We then conducted a national survey on smoking behavior to compare with the findings from the disaster area; hence, the national survey excluded the three disaster-hit prefectures (Iwate, Miyagi, and Fukushima). We randomly selected 2000 people by two-stage random sampling based on the points and the resident registers. We obtained answers from 1082 people (54% of response rate, 59% of real response rate; including one incomplete questionnaire). The survey period was from November to December 2013. The content of the questionnaire was similar to that of the 2012 survey conducted in the disaster area. The 2014 survey was conducted with the respondents of the 2012 survey, though funding limitations meant only half as many participants in the inland area could be interviewed. Thus, 982 people from the coastal area and 475 people from the inland area (total of 1457) were asked to take the second survey, and 577 (59% of response rate) and 353 people (74% of response rate) agreed to do so (a total of 930 respondents). The survey period was from November to December 2014. Many respondents who were registered in the coastal area in the 2012 survey could not be contacted in 2014 survey: 199 people had moved to unknown places; 40 people were absent for a long time, and the addresses of 19 people were not exist out of 982 coastal samples. The actual response rate for the coastal area was 80%, similar to the 84% for the inland area.

### Measures

The survey covered current smoking status, nicotine dependence (FTND, Fagerström Test for Nicotine Dependence; TDS, Tobacco Dependence Screener) [12, 13], damage or challenges due to the situation during and after the tsunami and earthquake, and sociodemographic factors. Smoking status was classified into three groups (current smoker, ex-smoker, never smoker) according to the two questions: “Have you ever smoked a conventional cigarette more than 100?,” and “Did you every day or sometimes smoke cigarettes for this one month?”

FTND scale contains six items that evaluate the quantity of cigarette consumption, the compulsion to use, and dependence. Questionnaires are as follows. (1) How soon after you wake up do you smoke your first cigarette? (2) Do you find it difficult to refrain from smoking in places where it is forbidden? (3) Which cigarette would you hate most to give up? (the first one in the morning or any other cigarette), (4) How many cigarettes per day do you smoke? (5) Do you smoke more frequently during the first hours after waking than during the rest of the day? (6) Do you smoke when you are so ill that you are in bed most of the day? For scoring of FTND, yes/no items are scored from 0 to 1, and multiple-choice items are scored from 0 to 3. The items are summed to yield a total score of 0–10. The higher the total score, the more intense is the patient’s physical dependence on nicotine.

The indexes used for the analyses were the mean of FTND, the proportion of persons with moderate or severe nicotine dependence (scores higher four points) as per FTND, the proportion of persons with severe nicotine dependence (more than seven points) as per FTND, the mean of TDS, and the proportion of person with nicotine dependence as per TDS (more than five points). Exacerbation of the smoking status in the panel survey is that never smokers or ex-smokers become current smokers, and improvement of the smoking status is that current smokers become ex-smokers. AUDIT (Alcohol Use Disorders Identification Test) was used as a screening test for alcoholism [14]. It is said that the cut-off point of AUDIT varies from country to country, and studies conducted in Japan have used more than 12 points for problem drinking and more than 15 points for alcohol dependence [15].

### Data analysis

For statistical testing for means, the *t* test was used for the analysis, and the paired *t* test for the comparison of 2012 with 2014 results. For statistical testing for proportions, the chi-squared test was used to test the difference in proportion of 2012 and 2014. When the expectation numbers for chi-squared test are small, we used a Fisher’s exact test. We conducted multiple logistic regression analysis using the variable increase method by the likelihood ratio. We conducted multivariable analysis with smoking status or dependence status as a dependent variable. The dependent variable was current smoking in 2012 or 2014, and independent variables were coast area/inland area, sex, age, years of education, marriage status, employment status, and damage due to the disaster (as of 2012 or 2014). Because the association between candidate factors was strong especially for damage due to the disaster, the statistical analysis to examine each factor was repeated using a statistical model including sex, age, and each candidate factor. We analyzed data with personal information removed, using SPSS Ver. 24 (IBM SPSS, Chicago, IL, USA).

## Results

The current smoking rate in the coastal area affected by both the earthquake and the tsunami was 50.0% for male and 21.4% for female participants in 2012, higher than in the inland area affected by the earthquake only (34.7% for male and 7.6% for female). Smoking prevalence in the coastal area was higher than that in the nationwide survey in 2013 (31.2% for male and 10.6% for female) (Table 1).

**Table 1:**
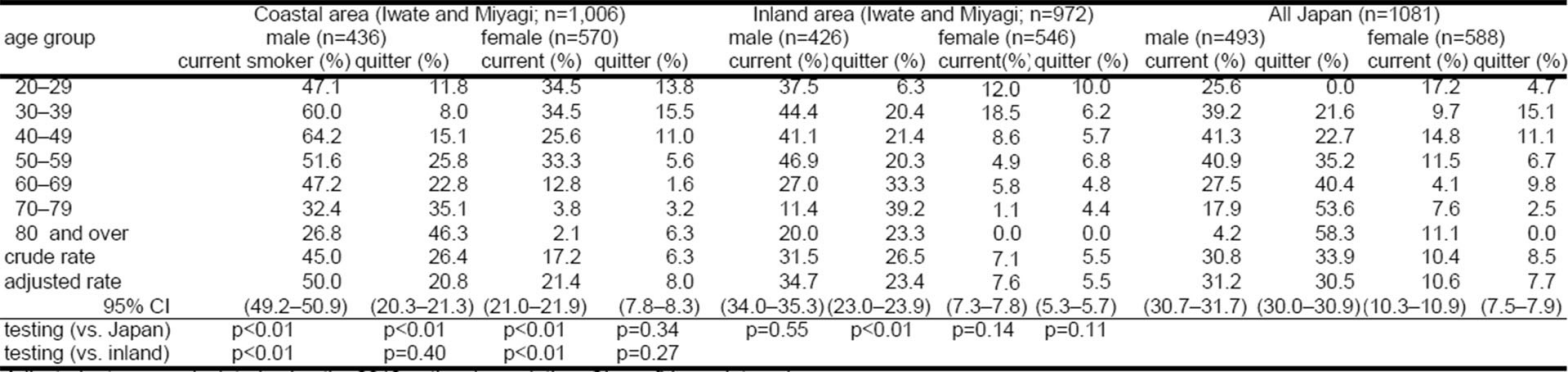
Smoking status by area (disaster are in 2012, all Japan in 2013)

Adjusted rate was calculated using the 2012 national population. *CI* confidence interval

The proportion of quitters in the coastal area in 2012 was 20.8% for male and 8.0% for female, whereas that in the inland area was 23.4% for male and 5.5% for female. These figures in coastal area were statistically similar to those in the inland area, and the figure for males in coastal area was statistically lower than the figure from a nationwide survey in 2013 (30.5% for male and 7.7% for female).

The prevalence of people with nicotine dependence in the coastal area in 2012 according to the FTND test was 8.7% for severely dependent males and 29.4% for moderately dependent males and 2.8% for severely dependent females and 11.0% for moderately dependent females, whereas that in the inland area was 4.4% and 16.1% for males and 0.6% and 3.9% for females. The figures in the coastal area were statistically higher than those in the inland area (Table 2); they were also significantly higher than those from the nationwide survey in 2013 (2.6% and 15.3% for males, and 0.3% and 3.4% for females).

**Table 2:**
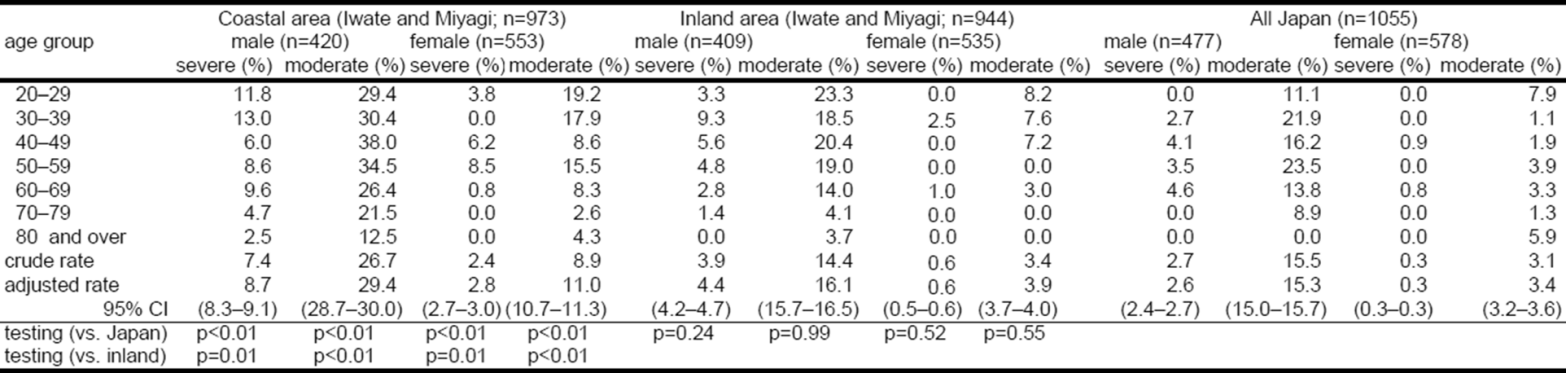
Nicotine dependence status by area (disaster area in 2012, all Japan in 2013)

Adjusted rate was calculated by using 2012 national population. Nicotine dependence status was assessed by FTND. *CI* confidence interval. Severe ≥ 7 points, moderate = 4–6 points

When we calculate change in smoking status between 2012 and 2014 among respondents to both surveys, respective rates of no change, improvement, and aggravation were 88.8%, 3.0%, and 8.2% for coastal males and 94.5%, 2.3%, and 3.2% for coastal females, while they were 96.2%, 1.3%, and 2.5% for inland males and 97.4%, 2.0%, and 0.5% for inland females. There were fewer persons with no change of smoking status in the coastal area compared with the inland area, and the rate of aggravation tended to be higher in coastal areas than in inland areas (Table 3).

**Table 3:**
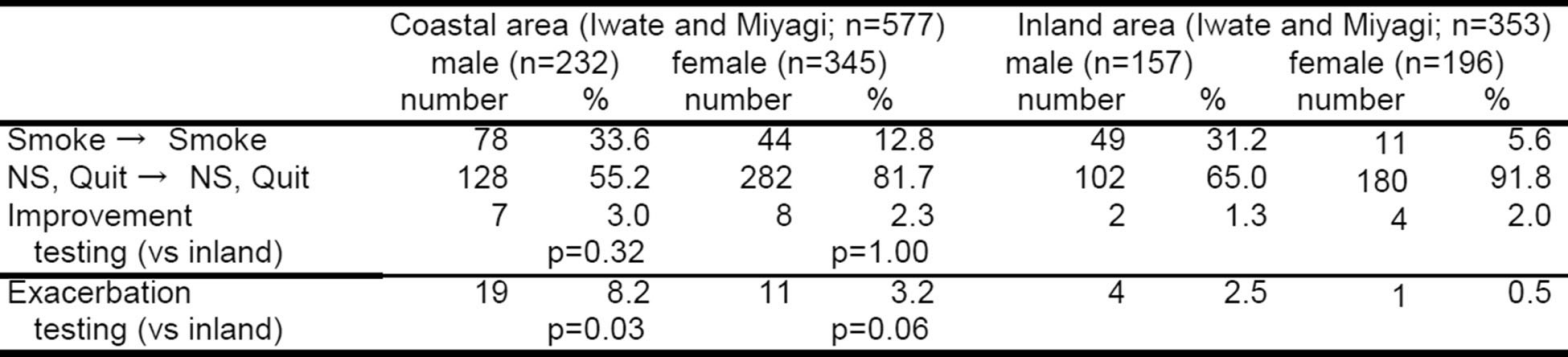
Change of smoking status from 2012 to 2014 (panel survey in disaster areas)

*NS* non-smoker

When we calculate change in nicotine dependence between 2012 and 2014 in the disaster area, we see no change, improvement, and aggravation at 84.7%, 9.4%, and 5.8% respectively for coastal males and 94.3%, 3.3%, and 2.4% for coastal females, whereas those figures are 84.8%, 5.7%, and 9.6% for inland males and 98.5%, 1.0%, and 0.5% for inland females. The rate of aggravation thus tended to be higher in coastal females and inland males (Table 4).

**Table 4:**
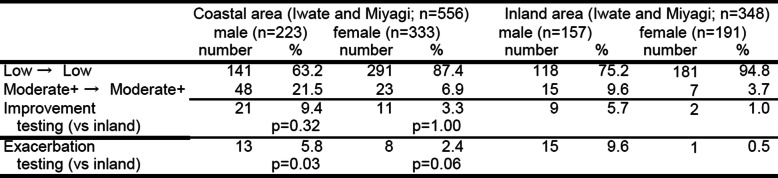
Change of nicotine dependence status from 2012 to 2014 (panel survey in disaster areas)

*NS* non-smoker, *Moderate+* moderate or severe

We applied multivariable analysis to examine factors associated with smoking behavior. In a statistical model explaining smoking status in 2012 (with covariates as of 2012), statistically significant risk factors for current smoking in 2012 were living in coastal area, divorce, under 9 years of education, unemployment, complete destruction of house, living in temporary housing, problem drinking (AUDIT score 12 points and over), and pathological gambling; a protective factor was professional agriculture, forestry, or fishery engagement (Table 5). The results of the multivariate analysis, which took into account the model fitness, showed that the complete destruction of houses was a significant risk factor.

**Table 5:**
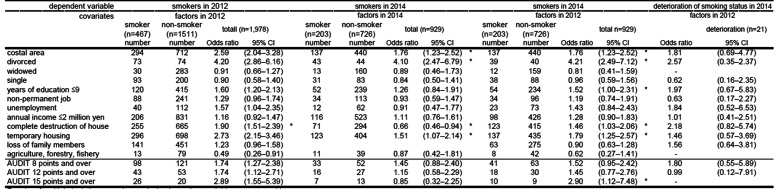
Factors associated with smoking status in 2012, 2014, and change of the status from 2012 to 2014

Results of multiple logistic regression analysis, dependent variable; current smoking. All covariates were adjusted by sex and age. Annual income means the individual income. *Statistically significant associated factors with good model fitness assessed by Homer-Lemeshow test

In the statistical model explaining smoking status in 2014 (with covariates as of 2014), significant risk factors for current smoking were coastal area, divorce, temporary housing, and prescribed drug dependence, while a protective factor was complete destruction of house. The results of the multivariate analysis, which took into account the model fitness, showed that the coastal area, divorce, complete destruction of houses, and temporary housing were significant risk factors.

In the statistical model to explain smoking status in 2014 with covariates as of 2012, the statistically significant risk factors were coastal area, divorce, under 9 years of education, complete destruction of house, temporary housing, and problem drinking (AUDIT score 15 points and over). In the statistical model explaining aggravation of smoking status in 2014 with covariates in 2012, the no statistically significant factors were found. However, the factors costal area, divorce, losing job by the disaster, complete destruction of house, temporary housing, and loss of family member tended to be risk factors for worsening smoking status. The results of multivariate analysis with the dependent variable replaced by nicotine dependence assessed by FTND or TDS were similar to these results (supplement tables).

## Discussion

This study has revealed the high smoking rate among both sexes in the tsunami disaster area after the great East Japan Earthquake and the low prevalence of male quitters in the area. The prevalence of nicotine-dependent people in the area was also higher than in the inland area or in other areas of Japan. The differences in prevalence were quite large. The high smoking rate and the rate of nicotine dependence in the coastal disaster area might have already been present in this area—coastal Tohoku region. Because there were no data before the earthquake disaster, we divided data from the nationwide survey in 2013 into respondents who lived under non-coastal and coastal local governments and compared the prevalence of smoking and nicotine dependence. There was no statistically significant difference in prevalence between these two areas. This suggests that the prevalence of smoking and nicotine dependence in the coastal area was elevated after the tsunami disaster and had not improved 3 years after the disaster. This health-related behavior may create and exacerbate future health problems in the disaster area.

Several articles on smoking behavior after disasters have been published. Smoking behavior before and after the 9/11 terrorist attacks was studied through a telephone survey, which found a higher smoking rate after the attacks [3]. In addition, low smoking cessation rate was reported among affected residents [4], rescue workers, and police officers with PTSD after the attacks [5]. It has been reported that high smoking rates and worsening of smoking status among residents are strongly related to posttraumatic stress disorder (PTSD) and major depression [16, 17].

High smoking prevalence among victims of Hurricane Katrina was also reported [6, 7]. A study on victims of Hurricane Katrina reported that PTSD and depressive symptoms are associated with smoking relapse [18]. There is a report that psychological distress after the disaster is not associated with worsening smoking behavior (increase of daily cigarette intake and nicotine dependence) [19]. In addition, the cigarette consumption was reported to increase after the Australian bushfires [8], while a follow-up study of the victims of the fireworks disaster reported that smoking became a predictor of mental disorder [20].

Previous articles related to change in smoking behaviors after an earthquake in New Zealand have been published; elevated smoking prevalence and nicotine dependence also were reported [10]. One article also claimed that there was a relationship between smoking behavior and PTSD (posttraumatic stress disorder) symptoms among Swiss victims of the Indian Ocean tsunami of 2004 [21]. As described above, there are many reports that the smoking prevalence and nicotine dependence of victims increases after natural disasters.

There are few articles reporting smoking behavior among victims by the tsunami after the great East Japan Earthquake. According to a report from Fukushima, few people started smoking after the disaster; the smoking rate was not high among victims in 2012 compared with smoking rate among the general population, and smoking rate decreased after the disaster [11]. A longitudinal study of elderly people in Iwate Prefecture from 2012 to 2015 found that smoking prevalence was higher among people with complete destroyed houses and that continued decreasing from 2011 through 2014 and increased in 2015 [22].

Although the Fukushima study was conducted on a large-scale, the response rate was low (41%); moreover, the study was cross-sectional rather than longitudinal. The Iwate study was limited to the elderly. The strengths of the present study are that it included the coastal, tsunami-hit area; participants were randomly sampled and included people aged 20 years and older; home visits were conducted for the survey interview; a nationwide survey was conducted for comparison; and some respondents were surveyed twice (longitudinally). Thus, this study was able to reveal changes in smoking behavior after a tsunami disaster, which worsened initially and did not improve 3 years later because of the protractedly damaged and difficult life situation that respondents were still facing due to the earthquake and tsunami. Although the results of current study are not similar to other reports from Japan, they are similar to results from other countries about smoking behavior after disasters.

There are reports that the prevalence of posttraumatic stress reaction and depressive reaction among resident survivors after the tsunami following the great East Japan Earthquake was high, and these symptoms were related to house flooding [23, 24]. There is a report that 3 years after the disaster, the depressive symptoms of survivors with loss of loved ones have recovered, but those have prolonged among survivors with property loss [25].

Therefore, it can be inferred that the high smoking rate and nicotine dependence observed in the current study have occurred through psychological distress due to damaged houses and long-term evacuation life caused by the tsunami. The smoking behavior may have been due to stress from crowding living conditions and interaction among the inhabitants of the temporary housing. Treatment for quitting thus be important for health care in temporary housing. In general, socioeconomic conditions, such as education level, income, and working conditions, are also related to smoking behavior [26, 27]. This study has revealed that the destruction of the house and subsequent temporary housing life after the disaster become important risk factors for smoking behavior even after adjusting for these socio-economic factors by multiple logistic regression analyses.

The present study has some limitations. First, the study participants did not include inhabitants of the Fukushima Prefecture. The sampling of participants from the Fukushima Prefecture was difficult because many evacuated people lived far away from their registered addresses. Second, the response rate relatively low. However, there are many inaccessible residents, and many people were exhausted from surveys by various researchers. Response rate of this survey was high one for surveys in the disaster areas because of the home visit interviews. Third, the follow-up survey was carried out only 2 years later. Since the research funds were limited, we could conduct the survey only twice. Fourth, the smoking status before the disaster is unknown; this is because survey was conducted after the disaster. We conducted a nationwide survey, excluding the three disaster prefectures, in 2013 using same survey methods and questionnaire, and compared the results with the results from the disaster areas, so that we were able to confirm that high smoking rate was a phenomenon persistent only in disaster areas.

## Conclusions

As shown in this study, smoking behavior and nicotine dependence worsened among victims of the tsunami disaster after the great East Japan Earthquake and had not improved after 3 years. In particular, the smoking behavior of inhabitants living in temporary housing is serious. The findings of this study stress the importance of improvising measures for smoking control in disaster areas in the long term to reduce future health hazards.

## Supplementary information

**Additional file 1.** Table a. Propotion of nicotine dependence accessed by TSD, by area (disaster area in 2012, All Japan in 2013). Table b. Change of nicotine dependence status accessed by TSD from 2012 to 2014 (Panel survey in disaster areas). Table c. Factors associated with nicotine dependence assessed by FTND score. Table d. Factors associated with nicotine dependence assessed by TDS score

## Data Availability

The datasets are not open to the public. Since all analyzes have not been completed, the consent from the members of this research group cannot be obtained.

## Abbreviations

Mw: Moment magnitude scale
FTND: Fagerström Test for Nicotine Dependence
TDS: Tobacco Dependence Screener
AUDIT: Alcohol Use Disorders Identification Test
CI: Confidence interval
NS: Non-smoker
Moderate+: Moderate or severe
PTSD: Posttraumatic stress disorder
