# Supplementary material for: Changes in smoking behavior among victims after the great East Japan earthquake and tsunami
Source: Environ Health Prev Med. 2020 Jun 11;25:19. doi: 10.1186/s12199-020-00858-5 (PMC7291441; doi:10.1186/s12199-020-00858-5)
Supplement: Supplementary file 1 — Additional file 1. Table a. Propotion of nicotine dependence accessed by TSD, by area (disaster area in 2012, All Japan in 2013). Table b. Change of nicotine dependence status accessed by TSD from 2012 to 2014 (Panel survey in disaster areas). Table c. Factors associated with nicotine dependence assessed by FTND score. Table d. Factors associated with nicotine dependence assessed by TDS score [file 12199_2020_858_MOESM1_ESM.pdf]

Table a. Propotion of nicotine dependence accessed by TSD, by area (disaster area in 2012, All Japan in 2013)

	Coastal area (Iwate and Miyagi; n=1,043)		Inland area (Iwate and Miyagi; n=935)		All Japan (n=1081)	
age group	male (n=455) (%)	female (n=588) (%)	male (n=407) (%)	female (n=528) (%)	male (n=493) (%)	female (n=588) (%)
20 ～ 29	17.6	15.4	16.7	10.2	2.6	10.9
30 ～ 39	13.0	19.6	27.8	8.9	20.3	3.2
40 ～ 49	26.0	17.3	22.2	4.3	18.7	4.6
50 ～ 59	19.0	15.5	15.9	1.0	19.3	4.8
60 ～ 69	21.6	5.0	15.0	3.0	11.0	3.3
70 ～ 79	15.0	3.3	6.8	1.1	4.8	1.3
80 and over	7.5	0.0	3.7	0.0	4.2	5.6
crude rate	7.5	2.4	3.9	0.6	2.6	0.3
adjusted rate	18.3	11.2	17.1	4.1	12.9	4.6
95% CI	(17.8 - 18.7)	(10.9 - 11.5)	(16.7 - 17.6)	(3.9 - 4.3)	(12.6 - 13.3)	(4.4 - 4.8)
testing (vs Japan)	p＜0.01	p<0.01	p=0.17	p=0.84		
testing (vs Inland)	p=0.13	p<0.01				

Adjusted rate was calculated by using 2012 national population. CI: confidence interval
Nicotine dependence status was assessed by TDS (5 points and over).

Table b. Change of nicotine dependence status accessed by TSD from 2012 to 2014 (Panel survey in disaster areas)

	Coastal area (Iwate and Miyagi; n=556)				Inland area (Iwate and Miyagi; n=348)			
	male (n=223)		female (n=333)		male (n=157)		female (n=191)	
	number	%	number	%	number	%	number	%
Dpend → Depend	23	10.3	13	3.9	16	10.2	5	2.6
No depend → No depend	170	76.2	296	88.9	125	79.6	180	94.2
Improvement	16	7.2	13	3.9	11	7.0	1	0.5
testing (vs inland)		p=0.36		p<0.01				
Exacerbation	14	6.3	11	3.3	5	3.2	5	2.6
testing (vs inland)		p=0.04		p=0.15				

Depend; dependence: TDS; Tobacco Dependence Scale

Table c. Factors associated with nicotine dependence assessed by FTND score

dependent variable	smokers in 2012			smokers in 2014			smokers in 2014			deterioration of smoking staus in 2014		
covariates	factors in 2012			factors in 2014			factors in 2012			factors in 2012		
	Odds ratio	95% CI		Odds ratio	95% CI		Odds ratio	95% CI		Odds ratio	95% CI	
costal area	3.03	(2.29–4.02)	*	2.05	(1.32–3.17)		2.05	(1.32–3.17)		0.95	(0.48–1.88)	
divorced	4.20	(2.82–6.27)		4.90	(2.86–8.40)	*	5.06	(2.88–8.89)	*	2.25	(0.86–5.93)	
widowed	0.78	(0.45–1.36)		1.28	(0.60–2.74)		1.47	(0.70–3.09)		3.19	(1.09–9.39)	*
single	0.90	(0.67–1.42)		0.66	(0.34–1.27)		0.97	(0.54–1.74)		0.31	(0.09–1.13)	
years of education ≤9	1.40	(1.01–1.95)		1.62	(1.00–2.60)	*	2.28	(1.41–3.69)		2.09	(0.94–4.66)	
non-permanent job	1.39	(0.99–1.95)		0.87	(0.50–1.52)		0.98	(0.55–1.74)		0.54	(0.16–1.85)	
unemployment	1.50	(0.94–2.38)		0.85	(0.37–1.93)		1.31	(0.70–2.46)		2.17	(0.85–5.51)	
annual income ≤2 million yen	1.26	(0.96–1.66)		1.33	(0.86–2.07)		1.55	(1.02–2.36)	*	1.26	(0.64–2.54)	
complete destruction of house	2.08	(1.59–2.71)	*	0.56	(0.36–0.85)	*	1.55	(1.03–2.33)	*	1.03	(0.52–2.03)	
temporary housing	3.17	(2.39–4.20)	*	1.75	(1.15–2.64)	*	2.08	(1.34–3.21)		0.97	(0.49–1.91)	
loss of family members	1.28	(0.97–1.69)					0.87	(0.57–1.32)		0.64	(0.29–1.39)	
agriculture, forestry, fishery	0.82	(0.43–1.56)		0.90	(0.38–2.11)		0.80	(0.32–2.00)		0.42	(0.06–3.17)	
AUDIT 8 points and over	2.40	(1.73-3.34)		1.59	(0.92–2.73)		1.40	(0.83–2.36)		1.11	(0.46–2.70)	
AUDIT 12 points and over	2.13	(1.35–3.36)		1.52	(0.74–3.11)		0.95	(0.44–2.07)		1.09	(0.31–3.78)	
AUDIT 15 points and over	3.84	(2.07–7.13)		0.94	(0.33–2.73)		2.10	(0.76–5.79)		1.83	(0.40–8.42)	

Results of multiple logistic regression analysis, dependent variable; moderate or severe nicotine dependence

*: statistically significant associated factors with good model fitness assessed by Hosmer-Lemeshow test

All covariates were adjusted by sex and age.

Table d. Factors associated with nicotine dependence assessed by TDS score

dependent variable	smokers in 2012			smokers in 2014			smokers in 2014			deterioration of smoking staus in 2014	
covariates	factors in 2012			factors in 2014			factors in 2012			factors in 2012	
	Odds ratio	95% CI		Odds ratio	95% CI		Odds ratio	95% CI		Odds ratio	95% CI
costal area	1.84	(1.36–2.50)		1.54	(0.96–2.47)		1.54	(0.96–2.47)		1.83	(0.86–3.91)
divorced	3.91	(2.56–5.98)	*	2.88	(1.60–5.20)	*	3.93	(2.16–7.17)	*	1.87	(0.73–4.79)
widowed	0.60	(0.30–1.19)		0.49	(0.16–1.44)		0.53	(0.18–1.56)		0.22	(0.03–1.75)
single	0.86	(0.56–1.33)		0.89	(0.45–1.74)		1.03	(0.54–1.95)		0.69	(0.24–1.97)
years of education ≤9	1.17	(0.80–1.73)		1.02	(0.58–1.80)		1.47	(0.85–2.56)		0.99	(0.41–2.38)
non-permanent job	1.63	(1.13–2.35)	*	0.81	(0.43–1.52)		1.18	(0.64–2.18)		1.34	(0.55–3.28)
unemployment	0.86	(0.46–1.61)		1.45	(0.66–3.20)		1.18	(0.56–2.49)		1.35	(0.46–3.98)
annual income ≤2 million yen	1.06	(0.78–1.45)		1.18	(0.72–1.95)		1.09	(0.68–1.74)		1.20	(0.58–2.45)
complete destruction of house	1.49	(1.10–2.00)	*	0.64	(0.40–1.03)		1.36	(0.86–2.15)		1.46	(0.72–2.98)
temporary housing	1.93	(1.42–2.62)		1.33	(0.84–2.09)		1.56	(0.98–2.51)		1.87	(0.88–3.97)
loss of family members	1.42	(1.04–1.95)		–			1.04	(0.65–1.67)		1.33	(0.66–2.69)
agriculture, forestry, fishery	0.59	(0.25–1.38)		0.49	(0.15–1.65)		0.17	(0.02–1.26)		–	
AUDIT 8 points and over	2.21	(1.53-3.19)		1.54	(0.84–2.83)		1.46	(0.81–2.62)		1.33	(0.51–3.47)
AUDIT 12 points and over	2.48	(1.53–4.05)	*	2.03	(0.95–4.30)		1.38	(0.61–3.14)		0.98	(0.22–4.33)
AUDIT 15 points and over	4.07	(2.17–7.63)	*	2.09	(0.77–5.69)		2.28	(0.79–6.61)		–	

Results of multiple logistic regression analysis, dependent variable; nicotine dependence

*: statistically significant associated factors with good model fitness assessed by Hosmer-Lemeshow test

All covariates were adjusted by sex and age.
